# Surveying Students and Alumni for Veterinary Curricular Renewal in a Portuguese Institution

**DOI:** 10.3390/ani15070986

**Published:** 2025-03-29

**Authors:** Joana C. Prata, Paula Proença, Paulo Martins da Costa

**Affiliations:** 1 School of Medicine and Biomedical Sciences, University of Porto (ICBAS-UP), Rua de Jorge Viterbo Ferreira 228, 4050-313 Porto, Portugal; pferreir@icbas.up.pt (P.P.); pmcosta@icbas.up.pt (P.M.d.C.); 2 Associate Laboratory i4HB, Institute for Health and Bioeconomy, University Institute of Health Sciences, CESPU, 4585-116 Gandra, Portugal; 3 UCIBIO Applied Molecular Biosciences Unit, Translational Toxicology Research Laboratory, University Institute of Health Sciences, CESPU, 4585-116 Gandra, Portugal

**Keywords:** veterinary curriculum, curricular renewal, higher education

## Abstract

The veterinary profession is rapidly evolving, requiring curricular renewal to address emerging challenges and enhance graduate preparedness. A survey was conducted among students and alumni of the School of Medicine and Biomedical Sciences of the University of Porto (*n* = 279), Portugal, to evaluate the need for an updated veterinary curriculum. Respondents conveyed a strong preference for companion animal medicine, likely influenced by sociocultural factors and media representation. To address current and future demands, it was recommended to re-evaluate curricular structures (e.g., course duration and electives), incorporate non-technical skills, and enhance practical education. Additional reforms should be supported by feedback mechanisms and stakeholder consultations. Alumni expressed dissatisfaction with the job market, which should be addressed through continuous education and specialization and greater intervention of the Portuguese Veterinary Board. In summary, areas for improvement and increased stakeholder involvement were identified as opportunities for veterinary curricular renewal.

## Simple Summary

Rapid changes in the veterinary profession create the need for schools to update their curricula. A survey of 279 students and alumni from the School of Medicine and Biomedical Sciences of the University of Porto in Portugal highlighted areas for improvement in the veterinary course. Many participants showed a strong interest in working with companion animals, influenced by culture and media. The survey helped to identify curricular improvements to better meet current and future needs, including extending course length, offering more elective options, teaching communication and other non-technical skills, and providing more hands-on training. Changes in the veterinary curriculum, informed by the opinions of students, graduates, and other stakeholders, will provide a better foundation for future veterinarians.

## 1. Introduction

The veterinary profession is experiencing a profound transformation, driven by societal shifts such as a decline in traditional animal production and an increased focus on food sciences, public health, and animal welfare [1,2,3]. These rapid developments and rising complexities create significant challenges for veterinarians in balancing their personal and professional roles [4]. Additionally, ongoing changes in production systems, evolving regulation, and advancements in medical practices contribute to the constant pressure on professionals [5]. Consequently, these pressures, combined with insufficient remuneration, contribute to significant dissatisfaction with the job market [6]. Moreover, European veterinarians have reported a high self-reported stress level (2.7/4), with 22% of professionals taking two weeks’ medical leave to recover from mental health issues (e.g., burnout and depression) in the last three years [7]. Most stress seems to arise from the social environment, namely, from interactions with animal owners, which requires a greater investment in communication and stress management education [8].

Current veterinary education faces several challenges, including insufficient practical training and a disconnect between theoretical knowledge and real-world application [9]. There is a growing need for incorporating new areas to address job market demands, potentially leading to an overburdened curriculum [10]. Revising the curriculum to meet these needs is crucial but challenging due to the strain on human resources [11] and disruption of existing structures [12]. Typically, curricular changes are informed by reference institutions (e.g., the World Organization for Animal Health) [13], faculty members [14], colleagues [15], employers [16], and feedback from students and alumni (i.e., graduates) [17].

In the European Union (EU), Directive 2005/36/EC, amended by Directive 2013/55/EU, establishes principles of professional qualifications across member states. Criteria for veterinary training were created to ensure that professionals acquire the knowledge and skills necessary for animal care, disease prevention, food safety, responsible medicine use, and compliance with EU laws. Regulations require a full-time study period of at least five years covering basic subjects (e.g., biology), basic sciences (e.g., anatomy), clinical sciences (e.g., pathology), animal production (e.g., husbandry), and food hygiene (e.g., inspection). Further recognition can be achieved by voluntarily submitting to the evaluation of the European Association of Establishments for Veterinary Education (EAEVE), an organization dedicated to improving and harmonizing veterinary education. EAEVE evaluates veterinary schools and provides assurance about the quality of veterinary education. In Portugal, veterinary medicine is also regulated by the Portuguese Veterinary Board (Ordem dos Médicos Veterinários) and overseen by the Ministry of Agriculture and Fisheries and the National Authority for Animal Health (Direção Geral de Alimentação e Veterinária).

The Integrated Master’s in Veterinary Medicine at the School of Medicine and Biomedical Sciences, University of Porto, Portugal, was established in 1994 and is accredited by the national Higher Education Assessment and Accreditation Agency (A3ES) and approved by the EAEVE. The course offers a multidisciplinary education, with a large focus on the “One Health” concept, with a 5.5-year program that includes a 6-month professional training period (details in Table A1, Appendix A). Despite curricular updates in 2006 and 2017, discrepancies between student expectations and job market requirements have become apparent. Consequently, the curriculum is now under review to develop strategies for improvement.

Engaging students and alumni in curricular renewal can effectively address current job market needs and promptly identify knowledge gaps [18]. Alumni, regardless of having studied under earlier versions of the curriculum, continue to reflect the institution’s strategies. Their professional achievements and experiences serve as a testament to the institution’s ability to equip graduates with the skills needed for success, while also offering valuable insights into the effectiveness of its educational programs.

This study aims to support the renewal of the veterinary curriculum by identifying key areas for enhancement, gathering feedback, and ensuring that it aligns with the evolving demands of the profession through a survey of students and alumni from the School of Medicine and Biomedical Sciences (ICBAS). Therefore, the specific objectives of the study were as follows: (i) to develop a survey that could be used to characterize and identify the needs of students and alumni; (ii) to collect and record data on the course’s student population for the first time; and (iii) to interpret these data to support curricular revisions at the institution.

## 2. Materials and Methods

### 2.1. Survey Development

A questionnaire was constructed based on the necessities of information for curricular renewal and on previous surveys conducted with veterinary students and professionals [19,20,21,22]. It included 41 questions in various formats, namely, multiple-choice, drop-down, Likert scale, ranking, and open-ended questions. These questions were divided into categories: 10 for participant characterization, 7 about ingress, 4 on areas of interest, 13 on alumni professional paths, 3 regarding opinions on classes, and 7 on emerging areas and curricular renewal. Open questions were optional for additional comments. Some questions were directed specifically to either students or alumni, based on initial responses.

### 2.2. Target Group and Recruitment

The study was targeted at students and alumni of the School of Medicine and Biomedical Sciences, University of Porto. The survey was conducted using LimeSurvey, hosted on the institution’s servers. The survey link was distributed to all the students via institutional email, while alumni received it through media, social media (e.g., Facebook and LinkedIn), and word of mouth. Data collection took place from 3 April 2024 to 2 June 2024.

Participants remained anonymous, except for students who provided their internal identification number for follow-up matching. To preserve anonymity, internal identification numbers were excluded from the present analysis. For this reason, the survey was promoted to ensure a representative number of responses from different groups without considering other personal aspects (e.g., gender). Based on the enrollment of 60 to 65 students per year, the study aimed at gathering responses from approximately 50% of students in each category and at least 100 alumni.

### 2.3. Data Analysis

Responses were exported as a Microsoft Excel file. Incomplete responses were removed from the database. Statistical analysis was conducted on IBM SPSS Statistics 29, including descriptive statistics, Fisher’s exact test, the Mann–Whitney U test, and the Kruskal–Wallis test, considering α = 0.05. Results were reported as means and standard deviations for numerical variables and as absolute frequencies for qualitative variables. Comments provided on open questions were organized by topics in a SWOT analysis. Additional data are provided in the Supplementary Materials. The study was approved by the joint Ethics Committee of ICBAS and the Centro Hospitalar Universitário de Santo António (CHUdSA) (2024/CE/P17(P426/2023/CETI)) and by the Data Protection Unit (Ref. UPD 0125/2023).

## 3. Results

### 3.1. Characterization of Participants

The survey received 279 responses, distributed among 160 current students and 119 alumni (Table 1). Age varied between 18 and 52 years, presenting statistically significant differences between categories (*p* < 0.001). Females comprised 79% of respondents, and almost all students were Portuguese (99%). Most were enrolled as ordinary students (80%), especially first-year students and alumni <5 years (*p* = 0.005). Students in the fifth and sixth years often benefited from their special status as athletes (*p* = 0.003) and as associative managers (*p* = 0.011). Participants originated mainly from urban environments (64%).

### 3.2. Application Process Details

Alumni generally concluded the course within 6 to 7 years (3–11 years; Table 2). Further information on the application process can provide more details on the students’ backgrounds and interests. In Portugal, ingress of ungraduated students in higher education is conducted through a national call based on high-school grades and exams, on individual preferences expressed as six course–institution pairs ordered by preference, and on the numerus clausus. Institutions have alternative ingress routes for (i) candidates over 23 years, (ii) graduates from other courses, (iii) international candidates, and (iv) students exchanging institutions.

Participants showed a strong preference for studying veterinary medicine, most choosing the veterinary course at ICBAS as their first option (65%) while also applying to veterinary courses in other institutions (60%). Students in the 2nd–4th year presented higher than expected enrollment through alternatives to national calls (*p* = 0.032; e.g., internal calls for candidates over 23 years).

Considering applications to other courses offered by ICBAS, 25% applied to other courses, while 14% still preferred veterinary medicine. ICBAS was likely preferred as the highest-ranked institution in veterinary medicine, with the last students enrolling with a grade of 172.8/200.0 in 2023’s call. Conversely, 11% of participants likely got into veterinary medicine after failing to get into medicine at ICBAS (last enrollment grade: 186.8/200.0), and 14% likely also applied to Aquatic Sciences (last enrollment grade: 153.5/200.0).

### 3.3. Expectations at Enrollment

Most participants had contact with veterinary medicine as clients, when accompanying animals to veterinary appointments (45%; Table 3). Significant differences were found for never having had contact with veterinary medicine before course enrollment (*p* = 0.021), which was higher than expected in alumni >5 years, and for the higher impact of media and social media (*p* < 0.001) on current students compared with alumni. Most participants (51%) envisioned practicing companion animal medicine. A higher proportion than expected of 5th–6th-year students and alumni <5 years envisioned working in companion animal (*p* = 0.002) and exotic animal medicine (*p* = 0.039). Only 12% of participants expressed not having a preferential area at enrollment. Interestingly, 84% of students admitted that their preferred area might change during the course, while only 52% of alumni reported this having occurred.

### 3.4. Alumni Expectations and Perceptions

**Figure 1:**
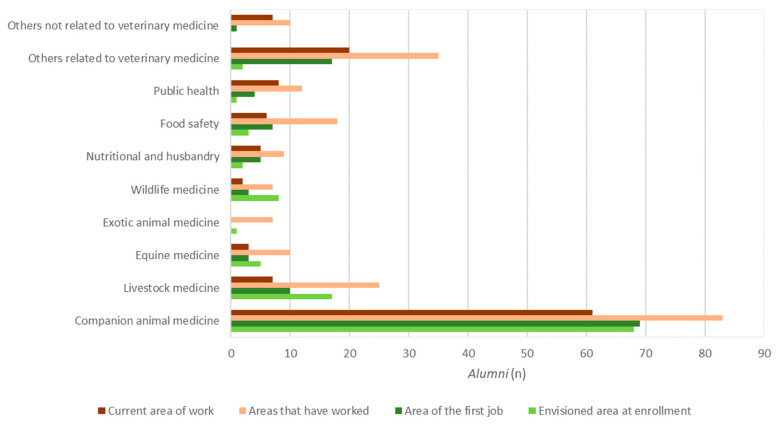
Areas of veterinary medicine practiced by alumni course conclusion compared to the envisioned areas at enrollment.

The most common areas of veterinary practice among alumni were companion animal medicine (51%), other areas related to veterinary medicine (17%; e.g., research, wildlife conservation, and pathological anatomy), and public health (7%). Clinical areas were generally overrepresented at enrollment compared to other areas of veterinary medicine (Figure 1). Mobility between areas of veterinary medicine was also evident.

Alumni reported finding a job within 3 months of course conclusion and generally having transitioned through two positions (Table 4), presenting a significant difference between having <5 years and >5 years of experience (*p* < 0.001). Most alumni reported working in the private sector (65%) in Portugal (84%) with full-time contracts (83%). Yet 61% reported that the job market did not meet their expectations. Other contract types included researchers (n = 3), other contracts for clinical practice (n = 2), internships (n = 1), PhD students (n = 1), and entrepreneurship (n = 1). Continuous education needs included post-graduations (66%), short courses (55%), and specializations (64%). Interest in post-graduation was significantly higher in alumni with >5 years of experience (*p* = 0.031).

Half of the alumni perceived theorical education as better at ICBAS (50%) compared to other national veterinary schools, compared to only 26% for practical education. Indeed, alumni reported as strengths the good theorical foundation; the high quality of practical classes; student experience gained at the Veterinary Teaching Hospital or by accompanying veterinary surgeons in ambulatory practice; the multidisciplinary One Health approach which involves sharing resources with other courses; the dedication and knowledge of faculty members; extensive training in companion animals, livestock, and aviculture; development of critical thinking skills; research being conducted at the institution; the availability of resources; improvement in practical capacities due to supervision and mandatory clinical acts; the opportunity offered by the final internship to hone competencies; and the high quality of veterinary surgeons from the institution.

**Figure 2:**
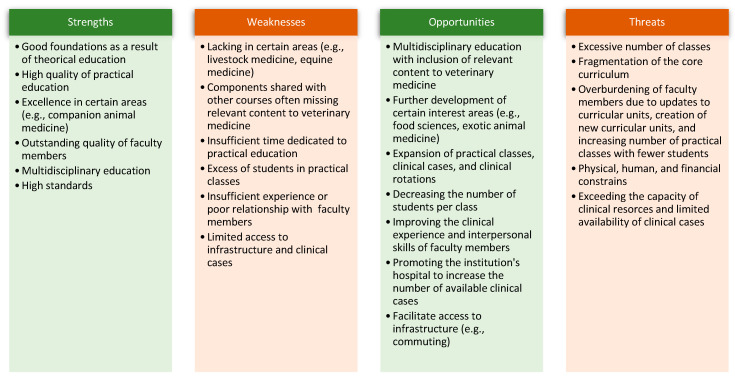
SWOT analysis of the quality of education at the institution based on alumni comments.

Areas of improvement were identified as the need for additional instruction on certain areas (e.g., food safety and technology, equine medicine, ovine and caprine livestock medicine, exotic medicine, wildlife, nutrition, oncology, vaccination, and imaging diagnosis), improving the veterinary component of curricular units shared between courses, increasing the number of experienced and/or specialized faculty members, increasing internship offers, cultivating the relationship between students and faculty members at the Veterinary Teaching Hospital, improving time devoted to practical skill learning, teaching to international standards (e.g., United Kingdom and United States standards), reducing the number of students in practical classes, increasing practical classes (including non-clinical areas) while reducing theorical–practical and laboratory classes, increasing practice under real-world conditions, and improving the number and diversity of appointments at the Veterinary Teaching Hospital. These comments were organized in a SWOT analysis presented in Figure 2.

### 3.5. Perceived Importance of Different Areas of the Veterinary Curriculum

**Figure 3:**
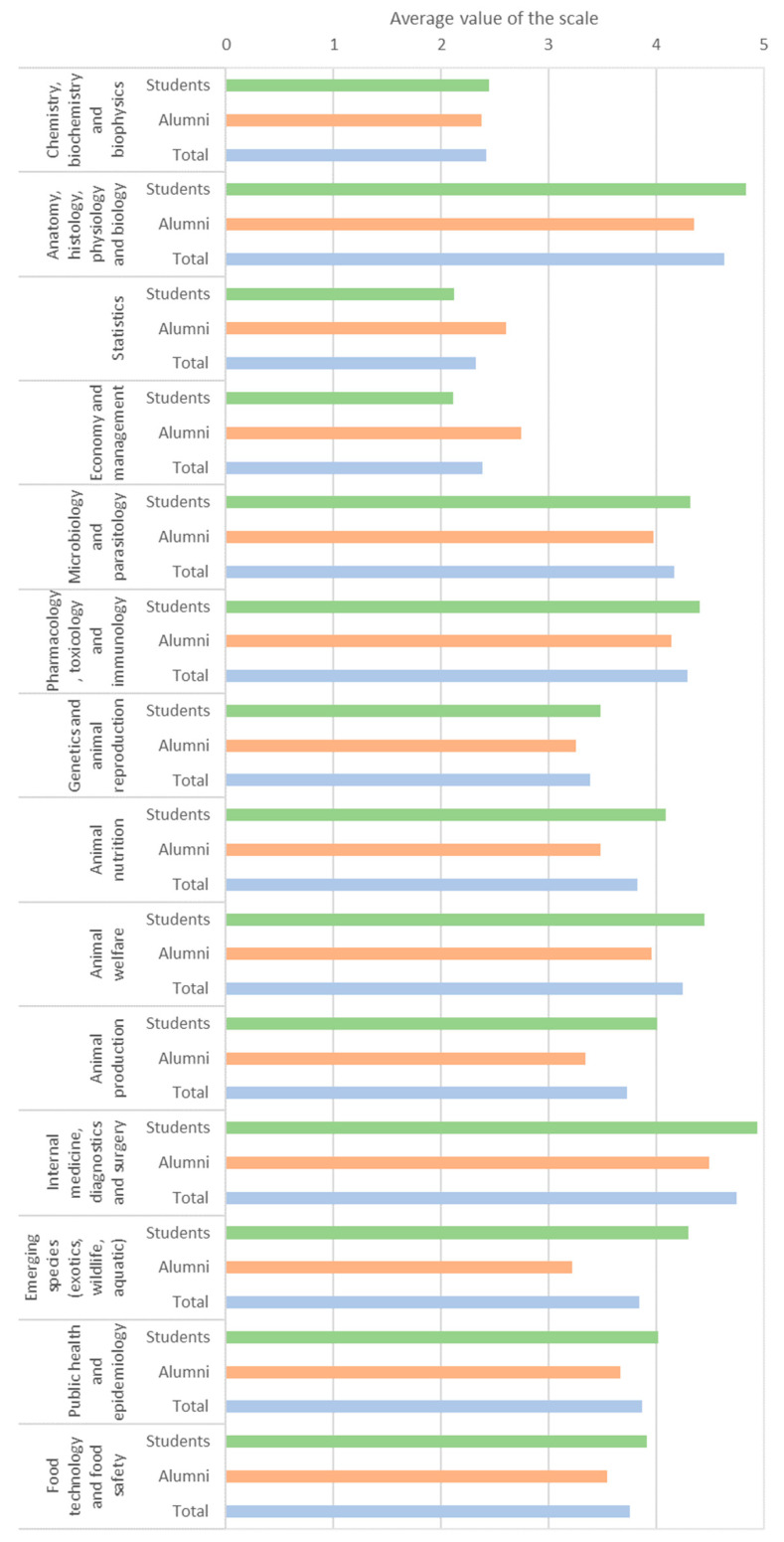
Average values attributed by participants to different areas of the veterinary curriculum (students in green, alumni in orange, and total in blue). The Likert scale represents the perceived relevance by the participants: none (1), low (2), average (3), high (4), extreme (5).

The three areas classified as of highest relevance were internal medicine, diagnostics, and surgery (4.8/5.0); anatomy, histology, physiology, and biology (4.6/5.0); and pharmacology, toxicology, and immunology (4.4/5). Conversely, the areas classified with lowest relevance were statistics (2.3/5.0); economy and management (2.4/5.0); and chemistry, biochemistry, and biophysics (2.4/5.0). Students generally presented higher variation in their responses compared to alumni (Figure 3).

### 3.6. Entities and Forces That Should Be Involved in Curricular Renewal

**Figure 4:**
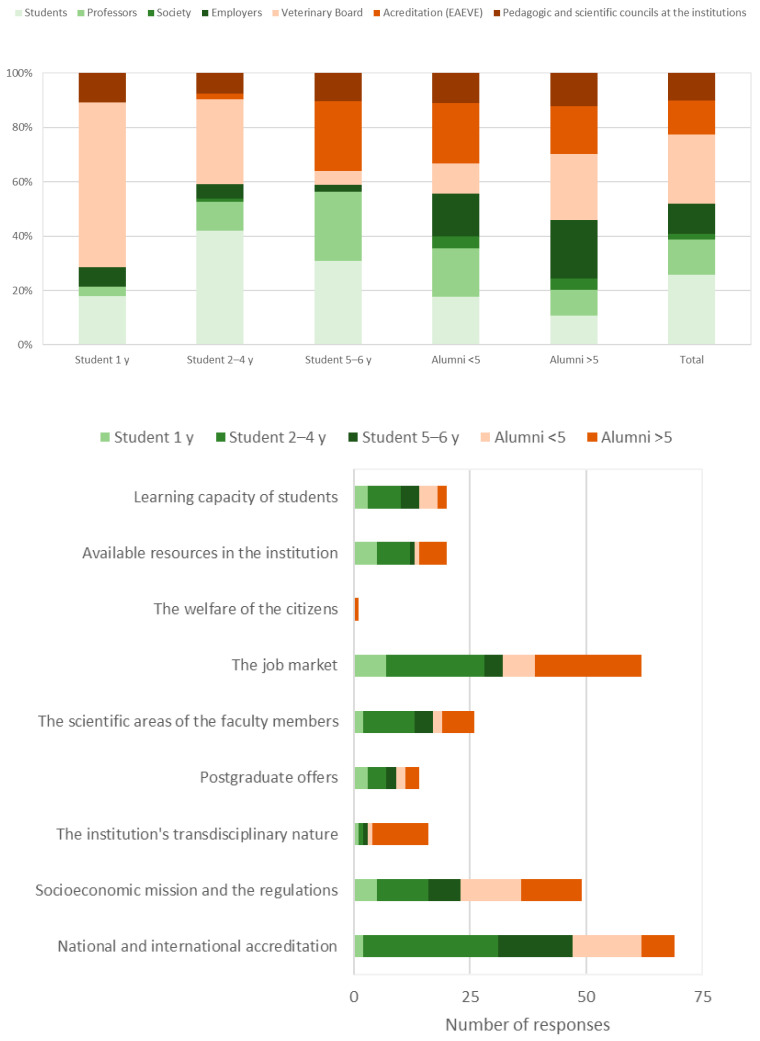
Principal entities that should be involved in curricular renewal (**top**) and main forces that should shape it (**bottom**) according to participants.

Participants identified students (25.8%), the Portuguese Veterinary Board (25.4%), and professors (12.9%) as the principal entities that should be involved in curricular renewal (Figure 4, top). However, this was not consensual among groups. For instance, the principal entity identified by students shifted from the Portuguese Veterinary Board in first-year students (60.7%) to students themselves in the following years (>30.8%). Accreditation entities gained relevance especially among 5th–6th-year students and alumni. Employers gained increasing importance among alumni (>15.6%). The main forces that should shape curricular renewal were identified as national and international accreditations agencies (24.9%), the job market (22.3%), and the socioeconomic mission and regulations of the profession (17.7%) (Figure 4, bottom).

### 3.7. Changes in Curricular Disciplinary Areas

**Figure 5:**
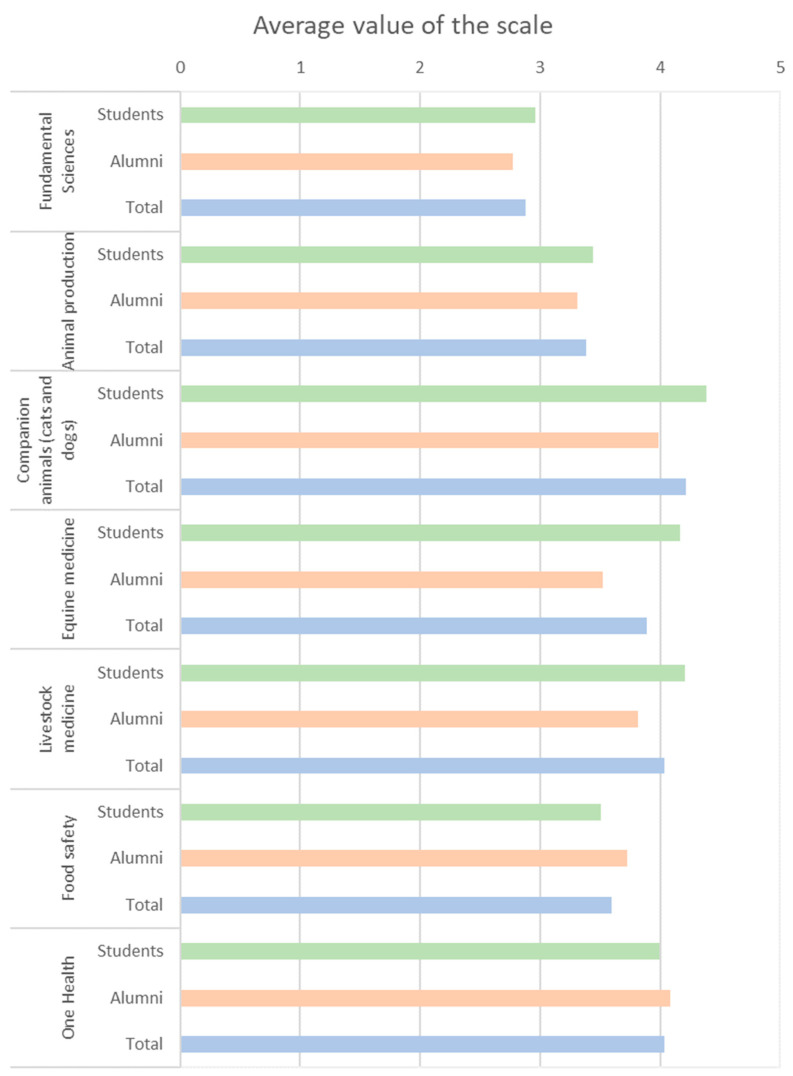
Relevance of further developing different disciplinary areas, according to participants (students in green, alumni in orange, and total in blue). The Likert scale represents the perceived relevance by the participants: none (1), low (2), average (3), high (4), extreme (5).

**Figure 6:**
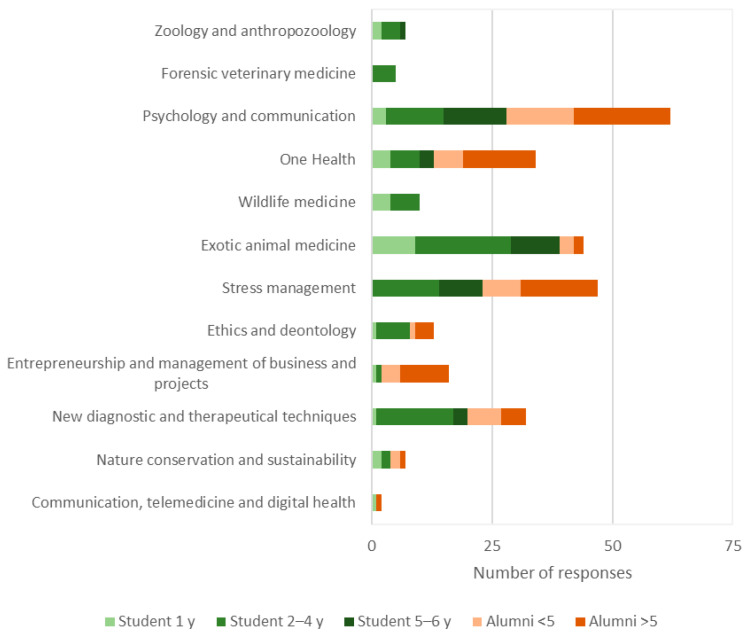
Competencies considered the most important for the veterinary profession and deemed to be included in the veterinary curriculum.

The disciplinary areas of higher relevancy for further development were companion animal medicine (4.2/5.0), equine medicine (4.2/5.0), and livestock medicine (4.0/5.0). Conversely, fundamental sciences were identified as the area with least relevance for further development (2.9/5.0) (Figure 5). Considering new competencies, participants identified psychology and communication (22.2%), stress management (16.8%), and exotic animal medicine (15.8%) as the most important to be included in the veterinary curriculum (Figure 6).

### 3.8. Pedagogic Strategies and Electives

**Figure 7:**
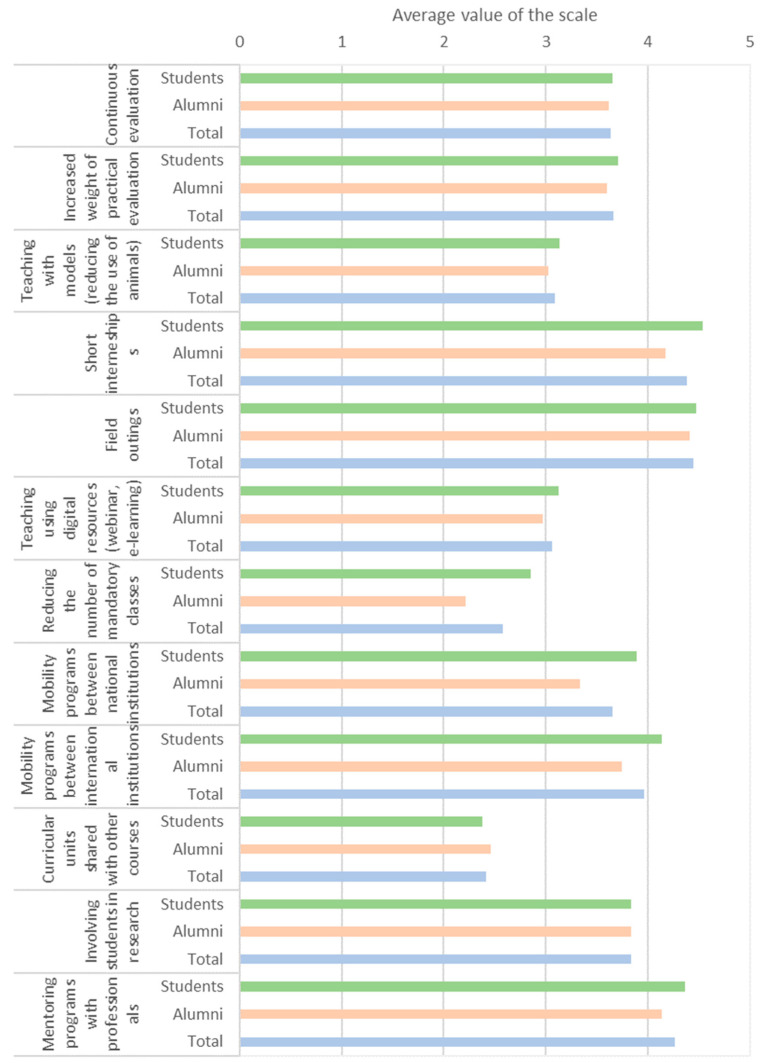
Relevance of each pedagogic strategy according to participants (students in green, alumni in orange, and total in blue). The Likert scale represents the perceived relevance by the participants: none (1), low (2), average (3), high (4), extreme (5).

Participants agreed that the veterinary course should have 12 semesters (6 years) instead of the current 11 semesters (5.5 years) (Table 5). Students and alumni both thought that two to three would be the ideal number of electives per semester. Moreover, they agreed that electives should begin in the third or fifth semester (first semester of second or third year, respectively). The groups only disagreed regarding beginning electives in the sixth or seventh semester (second semester of the third year and first semester of the fourth year), with a predominance of students and alumni, respectively. Finally, students identified the following pedagogic strategies as the most relevant: field outings (4.5/5.0), short internships (4.4/5.0), and mentoring programs with professionals (4.3/5.0) (Figure 7). Similar trends in responses were found for students and alumni.

## 4. Discussion

A survey was conducted to assess the status of and need for curricular renewal of the veterinary medicine course at the University of Porto, Portugal, collecting 279 responses. Most participants were female (79%), similar to the figures of 69% of active members of the Portuguese Veterinary Board [23] and 65% in other European countries [6]. Globally, women constitute the majority of students and veterinarians [24,25]. Yet there are still great disparities in earnings, service value, and practice ownership among women compared to men [24]. In the present work, no significant differences were found between sexes in regard to sector (*p* = 0.980) or type of contract (*p* = 0.652; Table S10). However, these responses do not suffice to determine whether Portuguese veterinarians experience gender discrimination, which was beyond the scope of this study.

The most common areas of veterinary practice amongst alumni were companion animal medicine (51%), other areas related to veterinary medicine (17%), and public health (7%). Similarly, most students anticipated working in companion animal medicine (47%). Interestingly, there was an underrepresentation of livestock medicine among the institution’s graduates, which is the second most common occupation in the country (11%), after companion animal medicine (65%) [23]. Conversely, students underestimated career paths involving other related areas (e.g., clinical pathology). Preferences may also depend on the cultural and socioeconomic environment, with previous studies showing that French students prefer exotic and wildlife medicine (23%) [26], American students in Texas prefer large/mixed-animal medicine (72%) [27], and Irish students prefer mixed practice (42.8%) [28]. Institutional orientation campaigns could be used to increase contact with these underrepresented areas and expand the students’ career options.

The preference for companion animal medicine among students and alumni likely stems from their sociocultural environment. Participants were mostly from urban environments (64%) and had previously been exposed to veterinary medicine as clients (45%). A greater influence of media and social media was observed for students compared to alumni. Students have a higher use of instant messaging and social media compared to veterinarians [29], and their career preferences can be influenced by mass media exposure [30]. National and international television series about veterinarians abound in Portuguese television. However, television series, being sentimental and entertainment-focused, may distort public perceptions of veterinarians by omitting many aspects of the profession [31]. This can also lead to the normalization of negative behaviors, affecting the development of professional skills [32]. Moreover, previous experience with animals and the area of residency can also influence species orientation [33]. This likely translated into a higher number of participants envisioning working with companion animals. Nonetheless, 84% of students and 52% of alumni still admitted that their preferences might change.

Most alumni reported not being satisfied with the job market (61%). This aligned with the low satisfaction with career choice (5.9/10), quality of life (5.1/10), work–personal life balance (4.6/10), and income (4.1/10) reported by Portuguese veterinarians [6]. The veterinary profession presents unique challenges, such as routine exposure to animal suffering and death, which translates into high burnout, compassion fatigue, and high suicide rates [34,35]. Additionally, the median annual income of a veterinarian working in Portugal is only EUR 19,200, below the European median of EUR 48,000 [6]. A study of German veterinary practitioners identified the key factors for work and life satisfaction as (i) good working atmosphere, (ii) reasonable salary, and (iii) holidays and leisure time [36]. More young Portuguese veterinarians are challenged by unsatisfactory earnings (59%) and poor work–personal life balance (52%) than by not having sufficient skills (31%) [6]. While institutions can improve skills training, it is equally crucial to tackle other challenges by incorporating psychology, communication, stress management, economics, management, and entrepreneurship into the curriculum. Interestingly, the current course on economy and management is among the lowest-rated in importance (2.4/5.0). Therefore, existing curricular units should be redesigned around the needs of veterinary professionals.

Despite discontentment with the career, alumni showed a keen interest in pursuing continuous education and/or specialization, even in the absence of continuing professional development (CPD) requirements. Portuguese veterinarians have previously reported that time (56%) and fees (67%) limit their ability to pursue CPD, with a preference for webinars (68%), congresses (65%), and online learning (61%) [6]. Interest in continuous education included post-graduations (66%), short courses (55%), and specializations (64%). Therefore, institutions should offer alternative learning methods, such as e-learning complemented by in-person sessions for practical classes, to better accommodate the needs of alumni. Moreover, courses for professionals should be outcome-based, focusing on learning needs and impact on professional development [37]. Indeed, CPD should employ teaching styles that help students achieve professional goals, promote community interaction, enhance critical thinking, and strengthen identity and wellbeing [38].

Alumni believed that ICBAS excels in theoretical education compared to other institutions, but they rated practical education less positively. Similarly, Portuguese veterinarians reported that veterinary schools do not train graduates with sufficient skills (6.5/10) [6]. Expanding practical education was a recurring theme throughout the responses. For instance, pedagogical strategies that garnered the most interest focused on practical education, including field outings (4.5/5.0), short internships (4.4/5.0), and mentoring programs with professionals (4.3/5.0). Similarly, areas related to clinical practice were the most popular, while fundamental sciences (especially outside biology) were the least. Indeed, fundamental sciences (e.g., physics) are frequently classified as irrelevant to the profession [5]. Moreover, veterinary students at James Cook University, Australia, expressed the desire for longer practical classes, particularly in earlier stages of the course [39]. There is a global trend for increasing the development of technical skills and clinical practice (either through case-based learning, simulations, or clinical rotations) while teaching fundamental sciences [12,15,40]. Students’ feelings of competency also increase with greater dedication to technical and professional education [25].

Regarding curricular renewal, participants agreed that the course should be expanded to 12 semesters (i.e., 6 years instead of 5.5 years) and that it should include two to three electives from the third to fifth semester onwards (i.e., second to third year). Interestingly, participants were interested in extending their education beyond the minimum training period of 5.5 years defined by Directive 2005/36/EC. The duration of veterinary courses varies between 5.5 and 6 years in Portugal and between 4 and 6 years in international institutions [41]. Electives provide opportunities to customize the curriculum and offer specialization in specific disciplines or species [12]. Priority areas to be included in the curriculum included psychology and communication (22%), stress management (17%), and exotic animal medicine (16%). This agrees with the need identified by European veterinarians to improve the curriculum on exotic animal medicine (63%) and communication skills (50%) [6]. Non-technical skills, including critical thinking, communication, and resilience, have become increasingly important in the veterinary profession [10,11]. Canadian veterinarians across various sectors (clinical practice, public sector, research, and corporate) rate non-technical skills, such as communication, teamwork, and critical thinking, as highly important, often scoring them 4 out of 4 in importance [42]. Moreover, a survey by the Iowa State University College of Veterinary Medicine found that non-technical skills contributed more to employer satisfaction than technical skills [43]. Thus, non-technical skills, particularly communication, should be incorporated into the veterinary curriculum [10]. Moreover, the presence of other biomedical courses at the institution, such as Medicine and Aquatic Sciences, benefited education by providing a shared One Health perspective.

Students (25.8%), the Portuguese Veterinary Board (25.4%), and professors (12.9%) were identified as the main entities that should be involved in curricular renewal, while national and international accreditation agencies (24.9%), the job market (22.3%), and the socioeconomic mission and regulations of the profession (17.7%) were identified as the main forces. Curricular renewals are needed to fulfill expectations in a rapidly changing profession [4]. Changes in curriculum often involve faculty members [14], colleagues from other veterinary schools [15], students [17], and alumni [16]. Interestingly, the Portuguese Veterinary Board was identified as a relevant entity, and it could contribute to clarifying the socioeconomic mission and regulations. Employers may also be asked to evaluate their satisfaction with new graduates [16], providing an insight into the needs of the job market. Regarding accreditation, participants most likely referred to the EAEVE, which establishes the standards for veterinary education and ensures international recognition of diplomas. While EAEVE provides a voluntary quality evaluation on education, veterinary courses must still follow Directive 2005/36/EC amended by Directive 2013/55/EU, as well as national agencies such as the A3ES and the Portuguese Veterinary Board. EAEVE helps define the minimum standards necessary for entry-level tasks in the veterinary profession, which are aligned with EU regulations. Students must be able to provide primary care to animals, maintain public health standards, uphold animal welfare, and navigate complex ethical and legal responsibilities. Moreover, they must be able to engage in lifelong learning and adapt to emerging challenges. Finally, engaging students in curricular renewal is crucial as it enhances transparency, addresses vocational gaps, and allows early identification of deficiencies in knowledge, skills, and market demands [18]. A summary of the key findings of the study is presented in Table 6.

## 5. Conclusions

The veterinary profession is evolving rapidly, marked by a decline in veterinarians working in animal production and an increase in emerging diseases, food sciences, public health, environmental protection, animal welfare, and clinical specialization [1,2]. Moreover, there is increasing female representation in the profession, and job market dissatisfaction is prevalent among Portuguese veterinarians. Therefore, renewal of veterinary curricula is required to fulfill the needs and expectations of the profession [4].

Both students and alumni show a strong preference for companion animal medicine, likely influenced by sociocultural factors, media representation, and the job market. To address current needs and future demands of the profession, both groups consider that curricular renewal should include the following: (i) extending the course duration to 12 semesters; (ii) incorporating electives to allow for personalized and specialized education; (iii) prioritizing emerging areas (e.g., exotic animal medicine); (iv) integrating non-technical skills (e.g., communication); (v) leveraging the One Health perspective; and (vi) enhancing practical education and clinical contact.

Engaging students in curricular reform is crucial, and this should be promoted through continuous feedback mechanisms. Anonymous surveys could also be used to consult other stakeholders, including faculty members and employers. For instance, students at the University of Minnesota College of Veterinary Medicine participate in weekly surveys on course progression, wellbeing, and perspectives to support curricular revisions [17]. Similarly, the University of Montreal’s Faculté de Médecine Vétérinaire evaluates the satisfaction of alumni and employers through surveys [16]. Implementing a continuous monitoring tool could support frequent adjustments to the veterinary curriculum, ensuring that it remains responsive to changing professional demands.

Despite discontentment with the career, alumni are keen on pursuing continuous education and specialization, even in the absence of mandatory CPD requirements. Veterinary schools can contribute to career improvements by ensuring that students acquire the necessary technical and non-technical skills and by providing opportunities for professional development through continuous education suited to professional demands. Institutions should offer alternative learning methods, such as e-learning complemented by in-person sessions for practical classes, to better accommodate the needs of alumni.

The Portuguese Veterinary Board has a greater role in shaping the profession and addressing the challenges posed by the job market. Collaboration between veterinary schools and the board can help align educational outcomes with the needs of the job market and increase the value of the profession, thus enhancing the overall satisfaction and success of veterinary graduates.

In summary, the veterinary profession is evolving, requiring updates to veterinary curricula to better prepare graduates for emerging challenges. Key recommendations include rethinking curricular structures, introducing non-technical skills, and expanding practical education. While veterinary schools can drive improvements, collaboration with the Portuguese Veterinary Board is essential for the valorization of the profession. Finally, continuous feedback mechanisms are crucial for ongoing adjustments to the curriculum.

## Figures and Tables

**Table 1:** Characterization of participants in the survey about the perception of the veterinary medicine course offered by the School of Medicine and Biomedical Sciences, University of Porto, Portugal.

Variable		Total	Students (Academic Years)			Alumni (Years of Experience)	
			1st	2nd–4th	5th–6th	<5	>5
Age	Average ± SD **	28 ± 7.8	20 ± 2.1	23 ± 5.3	27 ± 5.9	28 ± 4.3	37 ± 4.8
Sex	Male	58	6	18	9	5	20
	Female	221	22	75	30	40	54
Nationality	Portuguese	277	28	91	39	45	74
	Other	2	0	2	0	0	0
Student status	Ordinary *	222	27	71	24	39	61
	Student–worker	29	1	12	3	6	7
	Student–athlete *	8	0	3	5	0	0
	Student–associative manager *	15	0	3	6	0	6
	Special needs	5	0	4	1	0	0
Previous area of residency	Urban	178	21	63	25	28	41
	Rural	47	6	17	2	6	17
	Mixed	54	2	13	12	11	16
Total		279	28	93	39	45	74

* *p* < 0.05; ** *p* < 0.001. Student status: ordinary: regular full-time students; student–worker: students engaged in an employment relationship with a reduced academic contact time and other benefits; student–athlete: students engaged in high-competition sports with priority in selecting schedules and justified leaves of absence on competition days; student–associative manager: students engaged in youth associations (e.g., Students’ Council) can benefit from justified leaves of absence; special needs: students with particular health needs, declared by a recognized specialist, can benefit from increased support in teaching and evaluation methods.

**Table 2:** Application process details of participants in the survey on the veterinary medicine course offered by the School of Medicine and Biomedical Sciences, University of Porto, Portugal.

Variable		Total	Students (Academic Years)			Alumni (Years of Experience)	
			1st	2nd–4th	5th–6th	<5	>5
Enrollment years	Ingress	1994–2018	na	na	na	2003–2018	1994–2014
	Conclusion	2001–2024	na	na	na	2010–2024	2001–2018
	Duration	6 ± 1.1	na	na	na	6 ± 1.0	7 ± 1.2
Order or application	1st	180	22	61	23	32	42
	2nd–4th	46	5	12	6	9	14
	5th–6th	22	1	5	3	2	11
	Others *	31	0	15	7	2	7
Applied to other courses in the same institution	Yes	70	11	23	7	8	21
If so, preferred veterinary	Yes	39	7	16	3	6	7
Applied to a veterinary course in other institution	Yes	167	16	53	25	29	44
If so, preferred the institution	Yes	140	16	40	22	27	35

* *p* < 0.05; na, not available.

**Table 3:** Expectations of participants during enrollment on veterinary medicine course offered by the School of Medicine and Biomedical Sciences, University of Porto, Portugal.

Variable		Total	Students (Academic Years)			Alumni (Years of Experience)	
			1st	2nd–4th	5th–6th	<5	>5
Previous contact with veterinary medicine	Never *	50	2	13	5	7	23
	Media and social media **	33	9	12	6	5	1
	As a client	126	10	41	16	21	38
	Internship	36	4	12	9	6	5
	Veterinarian in the family or close friends	34	3	15	3	6	7
Envisioned area of practice at enrollment	Companion animal medicine *	143	7	43	25	31	37
	Livestock medicine	29	4	5	3	3	14
	Equine medicine	23	4	10	4	2	3
	Exotic animal medicine *	14	3	8	2	0	1
	Wildlife medicine	26	6	11	1	4	4
	Nutritional and husbandry	2	0	0	0	1	1
	Food safety	3	0	0	0	0	3
	Others related to veterinary medicine	3	0	0	1	0	2
	Others not related to veterinary medicine	1	0	0	1	0	0
	Did not know	33	4	15	2	4	8
Did you envision that the area could change during the course?	Yes	135	24	79	32	na	na
Did the area change during the course?	Yes	62	na	na	na	21	41

* *p* < 0.05; ** *p* < 0.001; na, not available.

**Table 4:** Alumni’s perceptions of the quality of education and experience in the job market.

Variable		Total	Alumni (Years of Experience)	
			<5	>5
Sector of current profession	Public sector	16	3	13
	Private sector	77	33	44
	Independent worker	9	4	5
	Research and education	17	5	12
Country of work	Portugal	100	37	63
	United Kingdom	7	1	6
	Spain	3	3	0
	France	2	0	2
	Italy	2	1	1
	Ireland	1	1	0
	Germany	1	0	1
	Austria	0	1	1
	Norway	1	0	1
Type of contract	Full-time	99	37	62
	Part-time	8	1	7
	Intern/temp	3	3	0
	Unemployed	1	0	1
	Retired	0	0	0
	Other	8	4	4
Job market corresponded to expectations	No	72	27	45
	Yes	42	16	26
	Exceeded	5	2	3
Perception of quality of theorical classes compared to other institutions	Worse	9	3	6
	Equal	51	17	34
	Better	59	25	34
Perception of quality of practical classes compared to other institutions	Worse	32	12	20
	Equal	56	21	35
	Better	31	12	19
Interest in continuous education and/or graduated courses	Short courses	65	26	39
	Post-graduations *	79	35	44
	Specialization	64	29	35
	Master’s	16	7	9
	PhD	40	18	22
	Not interested	6	0	6
Time to first job (months)	Average ± SD	3 ± 6.6	3 ± 7.8	2 ± 5.9
Number of different jobs	Average ± SD **	2 ± 1.7	1 ± 0.9	3 ± 1.8

* *p* < 0.05; ** *p* < 0.001.

**Table 5:** Number of semesters that the veterinary course should have according to participants.

Variable		Students	Alumni	Total
Semesters (%)	10	11.4	17.7	14.0
	11	16.4	17.7	16.9
	12	61.3	51.2	56.8
	Does not know	11.4	13.4	12.2
Semester from which electives should begin (%)	1	7.5	13.4	10.1
	2	13.8	8.4	11.5
	3	32.1	24.4	28.8
	4	8.2	6.7	7.6
	5	22.0	29.4	25.2
	6 *	6.9	1.7	4.7
	7 *	6.9	14.3	10.1
	8	2.5	0.8	1.8
	9	0.0	0.0	0.0
	10	0.0	0.8	0.4
Number of elective curricular units	Average ± standard deviation	2.44 ± 1.52	2.76 ± 1.57	2.58 ± 1.55

* *p* < 0.05.

**Table 6:** Key findings and recommendations to support curricular renewal of the veterinary medicine course.

Area	Findings	Recommendations
Gender	Most veterinary students are female.	Improve gender balance in enrollment and ensure equal employment opportunities.
Career Preferences	Most students want to work in companion animal medicine.	Promote underrepresented areas of veterinary medicine and expand students’ career options.
Background	Most students are from urban backgrounds and are influenced by media.	Create outreach campaigns to improve general knowledge about the profession.
Job Market	There is low satisfaction with the job market.	Increase the resilience of students by improving soft skills and entrepreneurship.
Continuous Education	Alumni are highly interested in pursuing continuous education and specialization.	Provide outcome-based courses for graduates in accessible learning formats (e.g., online).
Quality of Education	The institution is rated highly for theoretical education but less positively for practical training.	Expand practical education through field outings, short internships, and mentoring programs.
Curricular Renewal	Students support a curriculum of 12 semesters with electives.	Introduce two to three electives, starting from the second or third year onwards.
Stakeholders	In addition to accreditation bodies, students, the Portuguese Veterinary Board, and professors were identified as key entities for curricular renewal.	Continuously collect feedback from stakeholders, including employers, to provide educational outcomes aligned with the needs of the market.

## Data Availability

Data available upon request.

## Supplementary Materials

The following supporting information can be downloaded at https://www.mdpi.com/article/10.3390/ani15070986/s1: Table S1. Survey on the students’ perceptions and expectations of the veterinary curriculum structure; Table S2. Areas of veterinary medicine practiced by alumni <5 years (<5 y) and >5 years (>5 y) after course conclusion compared to the envisioned areas at enrollment; Table S3. Alumni that envisioned studying in an area and their distribution to the most recent area; Table S4. Average relevancy attributed to different areas of the veterinary curriculum, following a Likert scale, with 5 being extreme relevancy; Table S5. Entities that should be involved in curricular renewal (%); Table S6. Forces that should shape curricular renewal (n); Table S7. Average relevancy attributed to different areas of the veterinary curriculum, following a Likert scale, with 5 being extreme relevancy; Table S8. Competencies considered the most important for the veterinary profession and deemed to be included in the veterinary curriculum (n); Table S9. Relevance of each pedagogic strategy according to participants, following a Likert scale, with 5 being extreme relevancy; Table S10. Comparison between women and men for the sector of current profession and the type of contract.

## Abbreviations

The following abbreviations are used in this manuscript:

CPD	Continuing professional development
ICBAS	School of Medicine and Biomedical Sciences
NA	Not available

## Appendix A

**Table A1:** Current curriculum (2024) of the course on veterinary medicine at the School of Medicine and Biomedical Sciences, University of Porto, Portugal.

Year	Semester	Semester (Cumulative)	Curricular Unit	Credits
1	1	1	Cell Biology	4
1	1	1	Biophysics	5
1	1	1	Animal Cytogenetics	2
1	1	1	Animal Ethology	4
1	1	1	Exognosis and Animal Management	3
1	1	1	Quantitative Methods	6
1	1	1	Biological Chemistry I	6
1	2	2	Agriculture and Ecology	2.5
1	2	2	Systematic Anatomy I	4.5
1	2	2	General Physiology	4.5
1	2	2	Molecular Genetics	4.5
1	2	2	Animal Histology and Embryology I	4.5
1	2	2	General Microbiology	4.5
1	2	2	Biological Chemistry II	5
2	1	3	Systematic Anatomy II	5
2	1	3	Biochemistry	5
2	1	3	Veterinary Physiology	5
2	1	3	Animal Genetics and Improvement	5
2	1	3	Animal Histology and Embryology II	5
2	1	3	Veterinary Microbiology	5
2	2	4	Clinical Anatomy	5
2	2	4	Economy and Business Management	4
2	2	4	Immunology	5
2	2	4	Animal Nutrition	5
2	2	4	Parasitology	5
2	2	4	General Pathology	5
2	2	4	Animal Welfare, Deontology, and Veterinary Ethics	1
3	1	5	Anatomical Pathology I	5.5
3	1	5	Pharmacology and Therapeutics I	4
3	1	5	Imagiology	5.5
3	1	5	Animal Production I	4
3	1	5	Surgical Semiology	5.5
3	1	5	Clinical Semiology—Companion Animals	5.5
3	2	6	Anatomical Pathology II	5.5
3	2	6	Anesthesiology	5.5
3	2	6	Pharmacology and Therapeutics II	4
3	2	6	Pathology and Clinics of Infectious Diseases I	5.5
3	2	6	Animal Production II	4
3	2	6	Clinical Semiology—Farm Animals and Equine	5.5
4	1	7	Surgery—Farm Animals and Equine	6.5
4	1	7	Epidemiology	4.5
4	1	7	Pathology and Clinics of Infectious Diseases II	5
4	1	7	Food Technology I	4.5
4	1	7	Veterinary Toxicology	4.5
4	1	7	Farm Animal and Equine Internal Medicine I	5
4	2	8	Surgery—Companion Animals	5
4	2	8	Pathology and Clinics of Parasitary Diseases	5
4	2	8	Public Health	4.5
4	2	8	Food Technology II	4.5
4	2	8	Farm Animal and Equine Internal Medicine II	5
4	2	8	Small Animal Internal Medicine	6
5	1	9	Sanitary Inspection I	5
5	1	9	Internal Medicine and Surgery of Companion Animals I	10
5	1	9	Internal Medicine and Surgery of Farm Animals and Equines I	10
5	1	9	Theriogenology I	5
5	2	10	Sanitary Inspection II	5
5	2	10	Internal Medicine and Surgery of Companion Animals II	10
5	2	10	Internal Medicine and Surgery of Farm Animals and Equines II	10
5	2	10	Theriogenology II	5
6	1	11	Professional Training Period	30
