# Supplementary material for: Surveying Students and Alumni for Veterinary Curricular Renewal in a Portuguese Institution
Source: Animals (Basel). 2025 Mar 29;15(7):986. doi: 10.3390/ani15070986 (PMC11988073; doi:10.3390/ani15070986)
Supplement: Supplementary file 1 [file animals-15-00986-s001.zip › animals-3500431-supplementary.pdf]

Supplementary Material

Table S1. Survey on the students' perceptions and expectations of the veterinary curriculum structure.

Informed consent	
This questionnaire was developed as part of the research project "Students' perceptions and expectations of the veterinary curricula", being carried out at the School of Medicine and Biomedical Sciences of the University of Porto, by a team led by the Directorate of the Integrated Master's in Veterinary Medicine (MIMV) of the School of Medicine and Biomedical Sciences of the University of Porto (ICBAS).<br><br>This study focuses on students and *alumni of* the MIMV, with the following objectives:<br><br>- To assess the perception and expectations of students and *alumni* about the MIMV study plan;<br><br>- Investigate the professional situation of *alumni*;<br><br>- Identify emerging areas in veterinary medicine.<br><br>Your participation in this questionnaire will be voluntary and you will be guaranteed anonymity and confidentiality. It will be used exclusively in this research and may be disseminated in the scientific community.<br><br>This study was approved by the joint Ethics Committee of ICBAS and the Centro Hospitalar Universitário de Santo António (CHUdSA) 2024/CE/P17(P426/2023/CETI)) and by the Data Protection Unit (Ref. UPD 0125/2023).<br><br>Thank you in advance for your participation!	☐ Agrees with the participation in the study
**Sociodemographic data**	
Sex	☐ Male<br>☐ Female<br>☐ Other
Nationality:	(country list)
Date of birth:	(year list)

What is your situation regarding the institution?	☐ 1st year student<br>☐ 2nd – 4th year student<br>☐ 5th – 6th year student<br>☐ *Alumni* with up to 5 years' experience<br>☐ *Alumni* with more than 5 years' experience
Internal number (students only)	(short answer)
As a student, what is/was your status?	☐ Ordinary<br>☐ Worker-student<br>☐ Student-athlete<br>☐ Association leader<br>☐ Student with special education needs<br>☐ International student
Interaction (prior to entering the course) with the practice of veterinary medicine:	☐ Never<br>☐ Media (series) or social networks<br>☐ In the context of a consultation with an animal<br>☐ Internship<br>☐ Family or close person in which someone is a veterinarian or carries out related activities
Socio-demographic environment (where you have predominantly lived):	☐ Urban<br>☐ Rural<br>☐ Mixed
**Ingress in the course**	
Year of entry:	(year list)
Year of graduation: (alumni only)	(year list)
Where did the integrated master's degree in veterinary medicine at the institution rank in your preferences for applying to higher education?	☐ 1st option<br>☐ 2nd option<br>☐ 3rd option<br>☐ 4th option<br>☐ 5th option<br>☐ 6th option
Have you applied to other higher education courses offered by the institution?	☐ Yes<br>☐ No
Did you choose the integrated master's degree in veterinary medicine at the institution? (yes in previous question only)	☐ Yes<br>☐ No
Did you apply for veterinary medicine at another higher education institution?	☐ Yes<br>☐ No
Did you choose the integrated master's degree in veterinary medicine at the institution? (yes in previous question only)	☐ Yes<br>☐ No
**Areas of interest in veterinary medicine**	
What area did you want to work in when you started veterinary school? (Choose one)	☐ Companion animal medicine<br>☐ Livestock medicine<br>☐ Equine medicine<br>☐ Exotic animal medicine<br>☐ Wildlife medicine<br>☐ Animal nutrition and production<br>☐ Public health<br>☐ Food sector<br>☐ Other areas related to veterinary medicine<br>☐ Other areas not related to veterinary medicine
If others, specify which ones:	(short answer)
Do you think the area you want to work in might change during the course? (student	☐ Yes<br>☐ No

only) / Did the area you planned to work in change during the course? (*alumni* only)	
***Alumni* characterization (*alumni* only)**	
Current or most recent field of work: (choose one)	☐ Companion animal medicine<br>☐ Livestock medicine<br>☐ Equine medicine<br>☐ Exotic animal medicine<br>☐ Wildlife medicine<br>☐ Animal nutrition and production<br>☐ Public health<br>☐ Food sector<br>☐ Other areas related to veterinary medicine<br>☐ Other areas not related to veterinary medicine
Area of the profession in which you started: (choose one)	☐ Companion animal medicine<br>☐ Livestock medicine<br>☐ Equine medicine<br>☐ Exotic animal medicine<br>☐ Wildlife medicine<br>☐ Animal nutrition and production<br>☐ Public health<br>☐ Food sector<br>☐ Other areas related to veterinary medicine<br>☐ Other areas not related to veterinary medicine
Area of the profession which you have worked: (select all that apply)	☐ Companion animal medicine<br>☐ Livestock medicine<br>☐ Equine medicine<br>☐ Exotic animal medicine<br>☐ Wildlife medicine<br>☐ Animal nutrition and production<br>☐ Public health<br>☐ Food sector<br>☐ Other areas related to veterinary medicine<br>☐ Other areas not related to veterinary medicine
Type of sector in which you work:	☐ Public sector<br>☐ Private sector, employed by someone else<br>☐ Private sector, self-employed<br>☐ Research and education
Country of practice:	(country list)
What is your current contractual relationship?	☐ Full-time<br>☐ Part-time<br>☐ Internship/Temporary<br>☐ Unemployed<br>☐ Retired<br>☐ Other
How long did it take you to find your first job after graduating (in months)	(number)
How many full-time jobs have you had in áreas relevant to veterinary medicine, including changes in position and/or employer?	(number)
Has the job market in veterinary medicine met your expectations?	☐ Did not meet expectations<br>☐ Met expectations<br>☐ Exceeded expecations
What is your perception of the quality of theoretical teaching at the institution compared to colleagues in the same field who have studied at other higher education institutions?	☐ With worst preparation<br>☐ With equal preparation<br>☐ With better preparation
Why?	(short answer)
What is your perception of the quality of practical teaching at the institution	☐ With worst preparation<br>☐ With equal preparation

compared to colleagues in the same field who have studied at other higher education institutions?	☐ With better preparation
Why?	(short answer)
What kind of continuing training or graduate education course would you be interested in attending?	☐ Short course<br>☐ Post-graduation<br>☐ Specialization<br>☐ Masters<br>☐ Doctorate<br>☐ I'm not interested
What is your opinion of the range of continuing education or graduate courses on offer at the institution?	☐ Insufficient supply<br>☐ Sufficient supply<br>☐ Abundant supply
**Opinion on the areas of study**	
Evaluate the relevance of the following areas of study to the practice of veterinary medicine based on your personal experience/perception:<br><br>Likert scale:<br><br>1. Not relevant<br><br>2. Slightly relevant<br><br>3. Relevant<br><br>4. Highly relevant<br><br>5. Extremely relevant	1. Chemistry, biochemistry, biophysics<br>2. Anatomy, histology, physiology and biology<br>3. Statistics<br>4. Economy and business management<br>5. Microbiology and parasitology<br>6. Pharmacology, toxicology and immunology<br>7. Genetics and animal production<br>8. Animal nutrition<br>9. Animal welfare<br>10. Animal production<br>11. Internal medicine, diagnostics and surgery<br>12. Emerging species (exotic, wild, aquatic)<br>13. Public health and epidemiology<br>14. Food technology and health inspection
**Emerging study areas**	
In your opinion, who should have the most decisive say in the reformulation of the veterinary curriculum? (Choose only one option)	☐ Students<br>☐ Professors<br>☐ Society<br>☐ Employers<br>☐ Portuguese Veterinary Board<br>☐ Higher education certification bodies (ex. A3ES, EAEVE)<br>☐ Scientific and pedagogical management bodies
In which of the professional fields should training be further developed?<br><br>Likert scale:<br><br>1. Not relevant<br><br>2. Slightly relevant<br><br>3. Relevant<br><br>4. Highly relevant<br><br>5. Extremely relevant	1. Fundamental sciences<br>2. Animal production<br>3. Interaction animal medicine (dogs and cats)<br>4. Equine medicine<br>5. Livestock medicine<br>6. Health inspection and food safety<br>7. One Health (zoonosis control, nature conservation, animal welfare, …)
How important do you consider to include the following competencies in the future curriculum of veterinary medicine education? (Rank 3, ordering them by preference)	1. Psychology and communication<br>2. Stress management<br>3. Entrepreneurship and business and project management<br>4. Ethics and deontology<br>5. Zoology and anthropozoology<br>6. Nature conservation and sustainability<br>7. Wildlife medicine<br>8. Exotic animal medicine<br>9. Veterinary forensics<br>10. Communication, telemedicine and digital health<br>11. New diagnostics and therapeutics

	12. One Health
Indicate your perception of the importance of the actions listed in veterinary medicine education that is integrative, of high quality and responds to the needs of the profession.<br><br>Likert scale:<br><br>1. Not relevant<br><br>2. Slightly relevant<br><br>3. Relevant<br><br>4. Highly relevant<br><br>5. Extremely relevant	1. Reinforcing the practical study program<br>2. Continuous evaluation throughout the semester<br>3. Higher weight of practical evaluation<br>4. Model-based training (minimizing the use of animals)<br>5. Short-term internships<br>6. Field trips (e.g., outpatient, monitoring farms)<br>7. New forms of teaching (e.g., webinars, e-learning)<br>8. Reducing the number of compulsory classes<br>9. Mobility between national universities<br>10. Exchange programs between foreign universities<br>11. Common curricular units with other courses (e.g., Medicine, Aquatic Sciences)<br>12. Mentoring programs with professionals
In your opinion, what are the main forces shaping the veterinary curriculum? (Rank 3, ordering them by preference)	1. The socioeconomic mission of veterinarians and regulations<br>2. National and international accreditation<br>3. Resource availability at the institution<br>4. The scientific areas of our teaching staff<br>5. The transdisciplinary nature of the institution<br>6. The volume of information assimilable by students<br>7. Existing and development postgraduate courses<br>8. The job market for veterinarians<br>9. The well-being of Portuguese citizens
How many optional curricular units should there be per semester?	☐ 0<br>☐ 1<br>☐ 2<br>☐ 3<br>☐ 4<br>☐ 5
From which semester should optional curricular units be introduced?	☐ 1 (1st year, 1st semester)<br>☐ 2 (1st year, 2nd semester)<br>☐ 3 (2nd year, 1st semester)<br>☐ 4 (2nd year, 2nd semester)<br>☐ 5 (3rd year, 1st semester)<br>☐ 6 (3rd year, 2nd semester)<br>☐ 7 (4th year, 1st semester)<br>☐ 8 (4th year, 2nd semester)<br>☐ 9 (5th year, 1st semester)<br>☐ 10 (5th year, 2nd semester)
How many semester should the course have?	☐ 10 semesters (5 years, 300 ECTS)<br>☐ 11 semesters (5.5 years, 330 ECTS)<br>☐ 12 semester (6 years, 360 ECTS)<br>☐ I don't know
Submit your survey	(submit button)

Table S2. Areas of veterinary medicine practiced by *alumni* <5 years (<5y) and >5 years (>5y) after course conclusion, compared to the envisioned areas at enrollment.

**Variable**	**Envisioned area at enrollment**			**Area of the first job**			**Areas that have worked**			**Current area of work**		
	**Total**	**<5y**	**>5y**	**Total**	**<5y**	**>5y**	**Total**	**<5y**	**>5y**	**Total**	**<5y**	**>5y**
Companion animal medicine	68	31	37	69	31	38	83	35	48	61	28	33
Livestock medicine	17	3	14	10	4	6	25	7	18	7	3	4

Equine medicine	5	2	3	3	1	2	10	3	7	3	2	1
Exotic animal medicine	1	0	1	0	0	0	7	0	7	0	0	0
Wildlife medicine	8	4	4	3	1	2	7	1	6	2	1	1
Nutritional and husbandry	2	1	1	5	0	5	9	1	8	5	1	4
Food safety	3	0	3	7	2	5	18	2	16	6	1	5
Public health	1	0	1	4	1	3	12	0	12	8	2	6
Others related to veterinary medicine	2	0	2	17	4	13	35	7	28	20	4	16
Others not related to veterinary medicine	0	0	0	1	1	0	10	2	8	7	3	4

Table S3. *Alumni* that envisioned studying in an area and their distribution to the most recent area.

			Most recent area									
Envisioned area	n	Success (%)	Companion animal medicine	Livestock medicine	Equine medicine	Exotic animal medicine	Wildlife medicine	Nutritional and husbandry	Food safety	Public health	Others related to veterinary medicine	Others not related to veterinary medicine
Companion animal medicine	68	64.7	44	2	0	0	1	2	3	4	10	2
Livestock medicine	17	23.5	5	4	0	0	0	1	1	1	3	2
Equine medicine	5	23.5	1	0	2	0	0	1	0	1	0	0
Exotic animal medicine	1	0	1	0	0	0	0	0	0	0	0	0
Wildlife medicine	8	12.5	3	0	0	0	1	0	1	1	1	1
Nutritional and husbandry	2	0.0	0	1	0	0	0	0	1	0	0	0
Food safety	3	0.0	1	0	0	0	0	1	0	0	1	0
Public health	1	100.0	0	0	0	0	0	0	0	1	0	0
Others related to veterinary medicine	2	100.0	0	0	0	0	0	0	0	0	2	0
Others not related to veterinary medicine	0	0.0	0	0	0	0	0	0	0	0	0	0
Did not know	12	na	6	0	1	0	0	0	0	0	3	2

Table S4. Average relevancy attributed to different areas of the veterinary curriculum, following a Likert scale with 5 being extreme relevancy.

Areas	Respondents	Average
	Students	2.44
	Alumni	2.38
Chemistry, biochemistry and biophysics	Total	2.42
	Students	4.83
	Alumni	4.35
Anatomy, histology, physiology and biology	Total	4.63
	Students	2.12
	Alumni	2.61
Statistics	Total	2.33
	Students	2.11
	Alumni	2.75
Economy and management	Total	2.38
	Students	4.31
	Alumni	3.97
Microbiology and parasitology	Total	4.17
	Students	4.41
	Alumni	4.13
Pharmacology, toxicology and immunology	Total	4.29
	Students	3.48
	Alumni	3.25
Genetics and animal reproduction	Total	3.38
	Students	4.08
	Alumni	3.48
Animal nutrition	Total	3.82
	Students	4.45
	Alumni	3.96
Animal welfare	Total	4.24
	Students	4.01
	Alumni	3.34
Animal production	Total	3.72
	Students	4.94
	Alumni	4.49
Internal medicine, diagnostics and surgery	Total	4.75
	Students	4.30
	Alumni	3.22
Emerging species (exotics, wildlife, aquatic)	Total	3.84
	Students	4.02
	Alumni	3.66
Public health and epidemiology	Total	3.87
	Students	3.91
	Alumni	3.54
Food technology and food safety	Total	3.75

Table S5. Entities that should be involved in curricular renewal (%).

Entity	Student (academic years)			*Alumni* (years of experience)		Total	Statistics (p-value)
	1st	2nd – 4th	5th – 6th	<5	>5		
Students	17.9	41.9	30.8	17.8	10.8	25.8	<0.001
Professors	3.6	10.8	25.6	17.8	9.5	12.9	0.053
Society	0.0	1.1	0.0	4.4	4.1	2.2	0.403
Employers	7.1	5.4	2.6	15.6	21.6	11.1	0.004
Portugues Veterinary Board	60.7	31.2	5.1	11.1	24.3	25.4	<0.001
Accreditation agencies	0.0	2.2	25.6	22.2	17.6	12.5	<0.001
Pedagogic and scientific councils at the institutions	10.7	7.5	10.3	11.1	12.2	10.0	0.869

Table S6. Forces that should shape curricular renewal (n).

Force	Students (academic years)			*Alumni* (years of experience)		Total
	1st	2nd – 4th	5th – 6th	<5	>5	
National and international accreditation	2	29	16	15	7	69
Socioeconomic mission and the regulations	5	11	7	13	13	49
The institution's transdisciplinary nature	1	1	1	1	12	16
Postgraduate offers	3	4	2	2	3	14
The scientific areas of the faculty members	2	11	4	2	7	26
The job market	7	21	4	7	23	62
The welfare of the citizens	0	0	0	0	1	1
Available resources in the institution	5	7	1	1	6	20
Learning capacity of students	3	7	4	4	2	20

Table S7. Average relevancy attributed to different areas of the veterinary curriculum, following a Likert scale with 5 being extreme relevancy.

Areas	Respondents	Average
	Students	2.96
	Alumni	2.77
Fundamental Sciences	Total	2.88
	Students	3.44
	Alumni	3.31
Animal production	Total	3.38
	Students	4.39
	Alumni	3.98
Companion animals (cats and dogs)	Total	4.22
	Students	4.17
	Alumni	3.52
Equine medicine	Total	3.89
	Students	4.21
	Alumni	3.82
Livestock medicine	Total	4.04
	Students	3.50
	Alumni	3.72
Food safety	Total	3.60
	Students	3.99
	Alumni	4.08
One Health	Total	4.03

Table S8. Competencies considered the most important for the veterinary profession and deemed to be included in the veterinary curriculum (n).

**Areas**	**Students (academic year)**			***Alumni* (years of experience)**	
	**1st**	**2nd – 4th**	**5th – 6th**	**<5**	**>5**
Communication, telemedicine and digital health	1	0	0	0	1
Nature conservation and sustainability	2	2	0	2	1
New diagnostic and therapeutical techniques	1	16	3	7	5
Entrepreneurship and management of business and projects	1	1	0	4	10
Ethics and deontology	1	7	0	1	4
Stress management	0	14	9	8	16
Exotic animal medicine	9	20	10	3	2
Wildlife medicine	4	6	0	0	0
One Health	4	6	3	6	15
Psychology and communication	3	12	13	14	20
Forensic veterinary medicine	0	5	0	0	0
Zoology and anthropozoology	2	4	1	0	0

Table S9. Relevance of each pedagogic strategy according to participants, following a Likert scale with 5 being extreme relevancy.

Strategy	Respondents	Average
	Students	3.65
	Alumni	3.61
Continuous evaluation	Total	3.64
	Students	3.71
	Alumni	3.60
Increased weight of practical evaluation	Total	3.66
	Students	3.13
	Alumni	3.03
Teaching with models (reducing the use of animals)	Total	3.09
	Students	4.54
	Alumni	4.18
Short interneships	Total	4.38
	Students	4.47
	Alumni	4.41
Field outings	Total	4.45
	Students	3.13
	Alumni	2.97
Teaching using digital resources (webinar. e-learning)	Total	3.06
	Students	2.85
	Alumni	2.22
Reducing the number of mandatory classes	Total	2.58
	Students	3.89
	Alumni	3.34
Mobility programs between national institutions	Total	3.65
	Students	4.13
	Alumni	3.75
Mobility programs between international institutions	Total	3.97
	Students	2.38
	Alumni	2.46
Curricular units shared with other courses	Total	2.42
	Students	3.84
	Alumni	3.84
Involving students in research	Total	3.84
	Students	4.37
	Alumni	4.13
Mentoring programs with professionals	Total	4.27

Table S10. Comparison between women and men for the sector of current profession and the type of contract.

Variable		Female	Males	Statistics (p-value)
Sector of current profession	Public sector	13	3	0.980
	Private sector	61	16	
	Independent worker	7	2	
	Research and education	13	4	
Type of contract	Full-time	76	23	0.652
	Part-time	7	1	
	Intern/Temp	3	0	
	Unemployed	1	0	
	Retired	7	1	
